# Differentiating localized autoimmune pancreatitis and pancreatic ductal adenocarcinoma using endoscopic ultrasound images with deep learning

**DOI:** 10.1002/deo2.344

**Published:** 2024-03-02

**Authors:** Hitomi Nakamura, Motohisa Fukuda, Akiko Matsuda, Naohiko Makino, Hirohito Kimura, Yu Ohtaki, Yoshihito Nawa, Soushi Oyama, Yuya Suzuki, Toshikazu Kobayashi, Tetsuya Ishizawa, Yasuharu Kakizaki, Yoshiyuki Ueno

**Affiliations:** ^1^ Department of Gastroenterology Faculty of Medicine Yamagata University Yamagata Japan; ^2^ Department of Science Faculty of Science Yamagata University Yamagata Japan; ^3^ Institute of Well‐Being Yamagata University Yamagata Japan

**Keywords:** deep learning, diagnostic imaging, endoscopic ultrasound images, localized autoimmune pancreatitis, pancreatic ductal adenocarcinoma

## Abstract

**Objectives:**

Localized autoimmune pancreatitis is difficult to differentiate from pancreatic ductal adenocarcinoma on endoscopic ultrasound images. In recent years, deep learning methods have improved the diagnosis of diseases. Hence, we developed a special cross‐validation framework to search for effective methodologies of deep learning in distinguishing autoimmune pancreatitis from pancreatic ductal adenocarcinoma on endoscopic ultrasound images.

**Methods:**

Data from 24 patients diagnosed with localized autoimmune pancreatitis (8751 images) and 61 patients diagnosed with pancreatic ductal adenocarcinoma (20,584 images) were collected from 2016 to 2022. We applied transfer learning to a convolutional neural network called ResNet152, together with our innovative imaging method contributing to data augmentation and temporal data process. We divided patients into five groups according to different factors for 5‐fold cross‐validation, where the ordered and balanced datasets were created for the performance evaluations.

**Results:**

ResNet152 surpassed the endoscopists in all evaluation metrics with almost all datasets. Interestingly, when the dataset is balanced according to the factor of the endoscopists’ diagnostic accuracy, the area under the receiver operating characteristic curve and accuracy were highest at 0.85 and 0.80, respectively.

**Conclusions:**

It is deduced that image features useful for ResNet152 correlate with those used by endoscopists for their diagnoses. This finding may contribute to sample‐efficient dataset preparation to train convolutional neural networks for endoscopic ultrasonography‐imaging diagnosis.

## INTRODUCTION

Pancreatic ductal adenocarcinoma (PDAC) is one of the most aggressive types of cancer.^1^ By 2030, PDAC is expected to be one of the leading causes of cancer‐related deaths.^2^ Autoimmune pancreatitis (AIP) is a unique subtype of pancreatitis that is characterized by focal or diffuse swelling of the pancreas and narrowing of the pancreatic duct.^3^ AIP is classified into two types based on its pathogenesis. Most cases of AIP in Japan are type 1, which is the pancreatic manifestation of immunoglobulin G isotype 4 (IgG4)‐related disease.^4^ Among AIP cases, localized AIP with focal pancreas swelling mimics a pancreatic tumor. Hence, it is important to distinguish localized AIP from malignancies, especially PDAC.^5^ The International Consensus Diagnostic Criteria (ICDC) for AIP recommends pathological examination of localized AIP to avoid unnecessary surgery.^4^ Endoscopic ultrasound (EUS) is one of the most essential diagnostic procedures for evaluating pancreatic disease due to its excellent time and spatial resolution. However, the image diagnostic accuracy depends on the skill of endoscopists, who sometimes interpret EUS images subjectively. EUS‐guided fine needle aspiration/biopsy (EUS‐FNA/B) is a useful pathological diagnostic method for pancreatic lesions due to its high sensitivity and specificity.^6^ However, insufficient amounts of samples may affect the accuracy of the diagnosis. Furthermore, EUS‐FNA/B is invasive and may cause needle tract seeding.^7^, ^8^ Thus, objective and noninvasive methods to differentiate PDAC and localized AIP are desired.

Unlike conventional machine learning, deep learning is a machine learning technique of training a deep neural network (DNN), typically with a large amount of data, to automatically extract input features through hierarchical layers. It has improved the diagnostic accuracy of diseases in recent years.^9^, ^10^, ^11^, ^12^ Among DNNs, convolutional neural networks (CNNs) are specially designed for computer vision and a typical CNN consists of a classifier formed by a few fully connected layers and a feature extractor of many convolutional layers, which are comparable to the human visual cortex.^13^ In pancreatic diseases, CNNs using EUS images are effective in differentiating malignancies from intraductal papillary mucinous neoplasms^14^ and in detecting PDAC.^15^ As for AIP, CNNs applied to EUS images have accurately differentiated AIP from PDAC and other conditions.^12^ By contrast, we focused on tumor‐forming pancreatitis, such as localized AIP, which is rare and difficult to distinguish from PDAC. This naturally resulted in a small dataset, but we established a special cross‐validation framework to examine different data splits and verified some data splits are more efficient than others in training CNNs. Moreover, we observed that CNNs surpassed human experts with almost all data splits in various evaluation metrics.

## METHODS

### Patients

**FIGURE 1:**
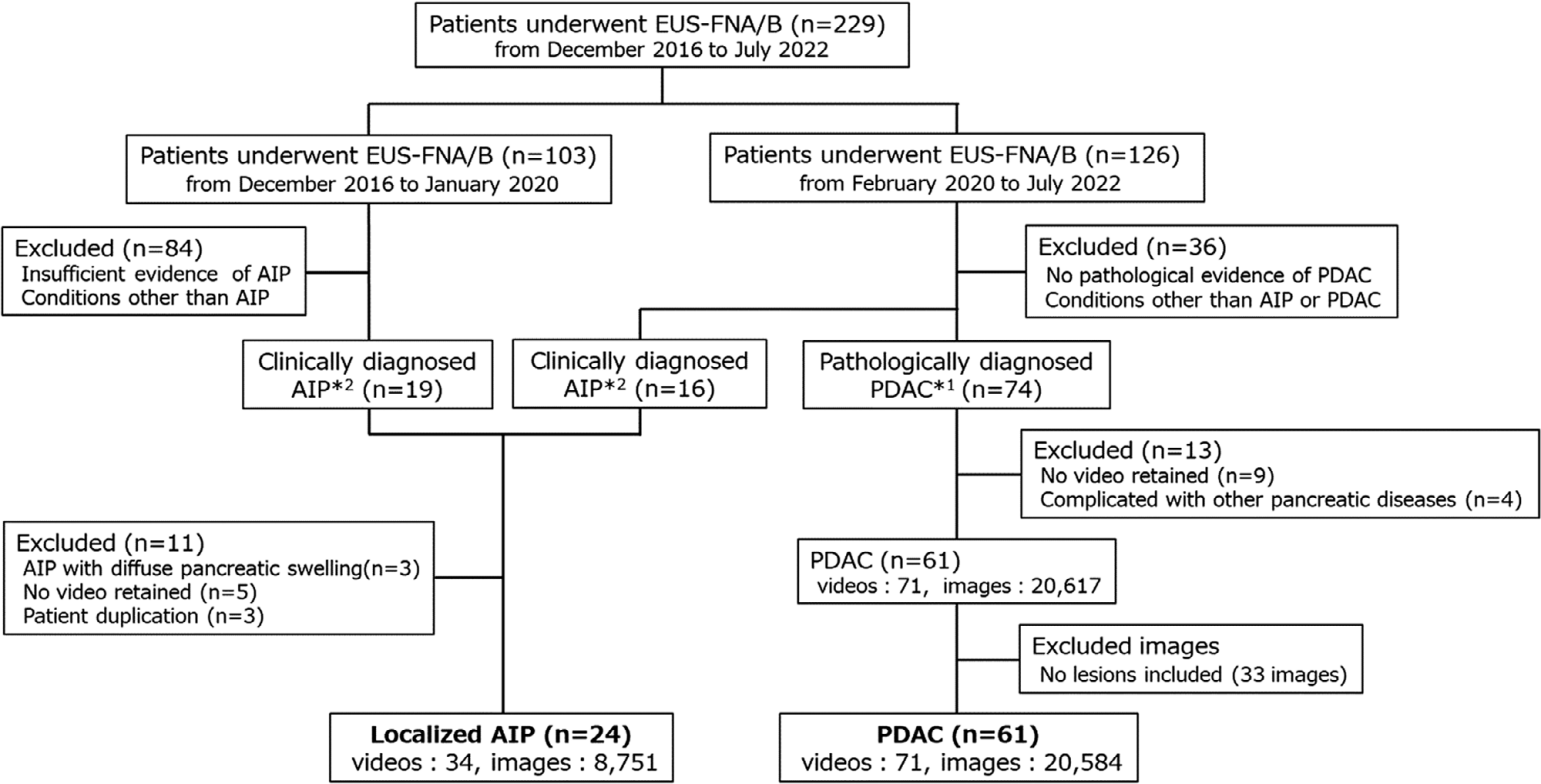
Flow diagram of endoscopy ultrasonography (EUS) image collection in this study. In total, there were 85 patients: 61 and 24 patients had pancreatic ductal adenocarcinoma (PDAC) and autoimmune pancreatitis (AIP) with localized pancreatic swelling, respectively, in whom endoscopic ultrasound‐guided fine needle aspiration/biopsy (EUS‐FNA/B) videos of pancreatic lesions were recorded. From these videos, all images of pancreatic lesions were saved as digital still images. Overall, 8751 and 20,584 EUS images of AIP and pancreatic ductal adenocarcinoma, respectively, were collected. Pancreatic ductal adenocarcinoma was pathologically diagnosed by either histological fragments obtained through EUS‐FNA/B, bile duct biopsy, or resected samples. AIP was diagnosed based on the International Consensus Diagnostic Criteria for AIP.

From February 2016 to July 2022, a retrospective study was performed on 229 consecutive patients who underwent EUS‐FNA/B. PDACs were histopathologically diagnosed based on histological fragments obtained by EUS‐FNA/B, bile duct biopsy, or resection. AIPs were diagnosed in accordance with the ICDC for AIP. Patients with pancreatic swelling less than 2/3 of the diagnosed AIP were included. Patients whose EUS‐FNA/B videos were recorded in a digital format were included in this study (Figure 1). The following features were obtained: age at the time of the EUS‐FNA/B, sex, body mass index (BMI), laboratory values (serum pancreatic amylase [P‐AMY], serum carcinoembryonic antigen [CEA], serum carbohydrate antigen 19‐9 [CA19‐9], and serum IgG4 levels), and amount of alcohol intake. We defined alcohol intake with reference to alcoholic and nonalcoholic fatty liver disease definitions as follows: light drinker, <20 g of ethanol/day; moderate, 20–60 g/day; and heavy, ≥60 g/day.^16^ This study was approved by the Institutional Review Board of the Yamagata University Faculty of Medicine (No. 2021–365, date: March 3, 2022).

### EUS procedure

On all patients, EUS‐FNA/B was performed using a Prosound F75 ultrasound system (Hitachi Aloka Medical) with a GF‐UCT260 curved linear echoendoscope (Olympus Corporation). Before inserting a biopsy needle, endoscopists observed the lesion and recorded a video clip for approximately 10 s. From these videos, all frames of the pancreatic lesions were stored as digital still images (JPEG format).

### Data preprocessing

**FIGURE 2:**
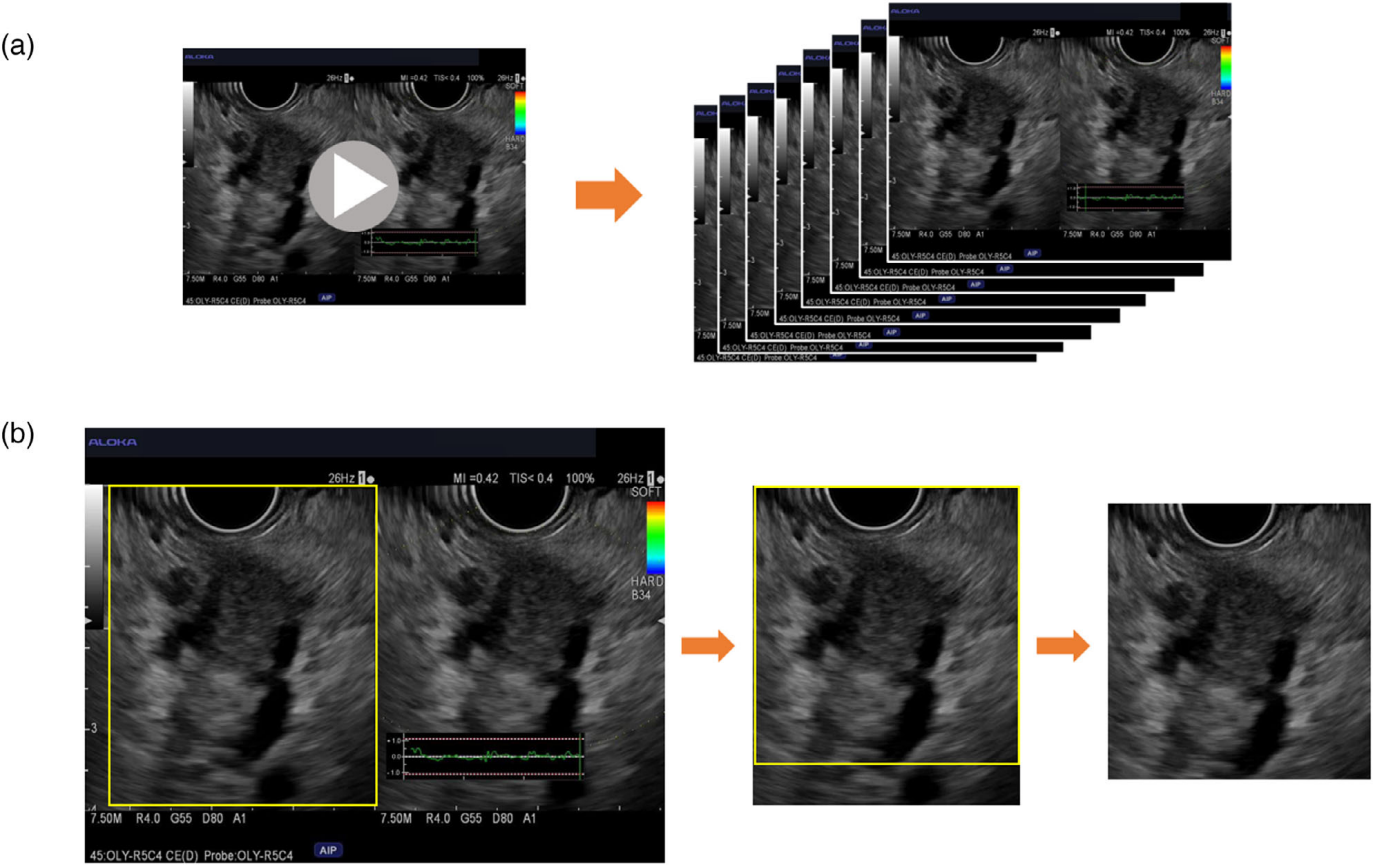
Endoscopy ultrasonography image preparation. (a) Prior to endoscopic ultrasound‐guided fine needle aspiration/biopsy of the pancreas, the lesions were observed and recorded for approximately 10 s. From these videos, all images of the pancreatic lesion were stored as digital still images (JPEG format) for each patient. (b) All endoscopic ultrasound images were uniformly cropped to remove potentially confusing image features and identifying information. This way, unbiased and anonymous images measuring 465 × 489 pixels constituted our dataset.

From the EUS videos, all frames of the pancreatic lesions were cropped to remove potentially confusing image features and identifying information. The process was uniform, and there was no room for biases in individual cases. More precisely, the bottom parts, where ultrasonic waves could not reach, were removed, and the biopsy needles, measuring ruler, doppler signal, and other features foreign to the original EUS image were excluded. This way, unbiased and anonymous images measuring 465 × 489 pixels were created and constituted our dataset (Figure 2).

### Dataset and cross‐validation

The images were split into training, validation, and test data for model evaluation. To avoid information leakage and to set up the cross‐validation framework, we divided patients into five groups (one test, one validation, and three training groups) instead of splitting all images. Note that multiple videos in a single EUS‐FNA/B session were obtained for some patients. Overall, we treated 20 (5 × 4) patterns of role allotments (test, validation, and training) for such individual groups. In particular, for each test group, there were four different choices for a validation group, yielding four differently trained CNNs. We applied the ensemble method and obtained the median of the predictions made by the four CNNs that served as the final prediction for the test data.

Since our dataset was small, random group division may contain biases. Hence, we first deliberately ordered the patients based on factors that may affect the performance of the model to overcome potential biases, which might sound contradictory. Then, for each factor we set the two group division manners and created the ordered and balanced datasets, to identify factors that have the greatest impact on the training of CNNs. Apart from the chronology of the sessions (CHR), considered a “vanilla factor,” we selected three factors from the patients’ data: age (AGE), self‐reported alcohol intake (SAI), and BMI. In fact, they influence the EUS image texture because of atrophy, fibrosis, and pancreatic and peripancreatic fat mass.^17^ Besides those four pairs, another pair of ordered and balanced datasets with respect to human diagnostic outcomes were created and are described in more detail below.

### Models

We employed the method of transfer learning because our dataset was small. A pretrained CNN of ResNet152,^18^ available via PyTorch,^19^ was mainly used in the current research, and only the last layer (the classifier) was replaced and trained while the other layers (the feature extractor) were frozen. Versions of ResNet with larger affixed numbers have deeper feature extractors and reportedly yield better performances.^18^ We trained ResNet50, ResNet101 and ResNet152 and compared their performances.

**FIGURE 3:**
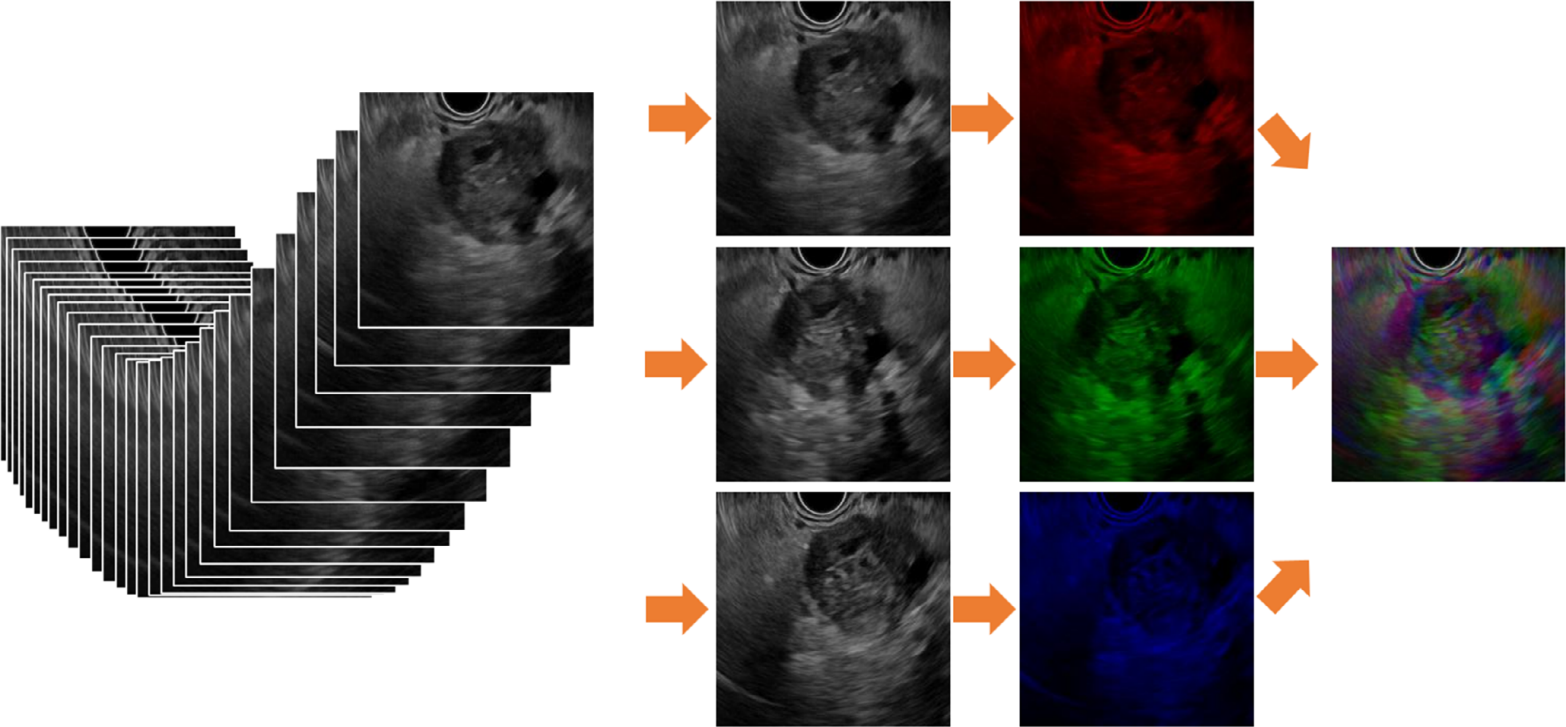
Quasi‐RGB images. Three grayscale images were randomly selected from endoscopic ultrasonography frames to create a quasi‐RGB image. A single quasi‐RGB image carries temporal data and contributes to data augmentation.

A unique approach in this study is that we fed quasi‐RGB images to CNNs. A quasi‐RGB image is comprised of three different grayscale frames of EUS videos that play roles in the three colors: red, green, and blue (Figure 3). Structurally CNNs separately intake those three colors, with which daily‐life images are described in slightly different manners. In quasi‐RGB images time‐wise differences are regarded as color‐wise differences, which is analogous to the “early version” method of applying CNNs to video classification,^20^ wherein several temporally different frames of a video are combined pixel‐wise to become inputs for CNNs. Moreover, it also contributes to data augmentation. They were resized to 224 × 224 before getting input into CNNs.

Our dataset contained more PDAC cases than AIP cases, which was unbalanced. To avoid imprinting such a bias to the CNNs, we generated about 1000 quasi‐RGB images almost evenly for AIP and PDAC during each training epoch; each quasi‐RGB image was made of video frames of the same patient. Each training session lasted for 20 epochs, and the model parameters that gave the best score for the area under the receiver operating characteristic curve (AUROC) with the validation data were stored for the later evaluation with the test data, wherein 300 quasi‐RGB images were randomly generated for each patient. Various data augmentations were applied during the training. As for hardware, Nvidia GPUs TITAN Xp and GeForce RTX 2080 Ti were used in this study.

### Endoscopists

Endoscopists were classified as experts or novices based on their qualifications granted by the Japan Gastroenterological Endoscopy Society. To compare the ability of CNNs with that of humans in differentiating localized AIPs from PDACs, seven endoscopists of our institution (four experts and three novices) not involved in dataset development diagnosed all cases in this study. They examined all EUS videos and made a diagnosis for each patient. Similar to the CNNs, the endoscopists were not provided with any additional information, such as the clinical course, laboratory data, or other data. However, they were allowed to watch the original EUS videos in any order and as many times as they wished. The scores of the novice and expert endoscopists were averaged separately or together. Thus, we introduced an additional pair of ordered and balanced datasets with respect to the endoscopists’ diagnostic accuracy (EDA).

### Statistical analyses

We assigned 0 and 1 to AIP and PDAC, respectively, and evaluated the CNNs using various metrics such as the AUROC, sensitivity, specificity, positive predictive value (PPV), negative predictive value (NPV), and accuracy. The threshold was set at 0.5 for all metrics except for the AUROC. We used AUROC as a representative indicator to evaluate efficient learning methods.

All tests were two‐tailed, and *p < 0.05* was considered statistically significant. Continuous variables were expressed as median and range. Fisher's exact test was used for categorical variables, and the Wilcoxon rank‐sum test was used for continuous variables.

## RESULTS

Among the 126 patients who underwent EUS‐FNA/B from February 2020 to July 2022, 74 were pathologically diagnosed with PDAC. Meanwhile, 61 patients with PDAC whose EUS videos (71 videos) were recorded in digital format were further investigated. From the videos, 20,584 frames of PDAC lesions were stored as digital still images. Among the 229 patients who underwent EUS‐FNA/B from December 2016 to July 2022, 35 patients were diagnosed with AIP. Meanwhile, 24 patients with localized AIP whose EUS videos (34 videos) were recorded in digital format were further investigated. From 34 videos, 8751 frames of AIP lesions were stored as digital still images (Figure 1).

### Characteristics of patients

The characteristics of patients are shown in Table 1. Serum IgG4 levels were significantly higher in patients with AIP than in those with PDAC, while CEA and CA19‐9 levels were significantly higher in patients with PDAC than in those with AIP (both *p < 0.05*). However, BMI, P‐AMY, and the amount of alcohol intake were not significantly different between the AIP and PDAC groups.

**TABLE 1:** Patients’ characteristics.

**Characteristics** **^†^**	**PDAC, *n* = 61**	**AIP, *n* = 24**	***p*‐value**
Age, years	71 (43–83)	67.5 (50–83)	0.725
Male sex	36 (59)	19 (79)	0.175
BMI^‡^, kg/m^2^	21.7 (15.9–30.3)	22.7 (19.1–37.5)	0.08
Laboratory values			
Serum P‐AMY, IU/L	26 (6–2464)	32 (6–409)	0.417
Serum CEA, ng/mL	4.04 (1.11–235.23)	2.01 (0.84–19.37)	0.016^*^
Serum CA19‐9, U/mL	608.7 (2–12,000)	4.15 (2–78.8)	<0.001^**^
Serum IgG4, mg/dL	38.5 (6–170)	499 (143–2,360)	<0.001^**^
Alcohol intake^§^			
Light	38 (62.3)	18 (75)	0.266
Moderate	18 (29.5)	5 (20.8)	0.4177
Heavy	5 (8.2)	1 (4.2)	0.5138

Abbreviations: AIP, autoimmune pancreatitis; CA19‐9, carbohydrate antigen 19‐9; CEA, carcinoembryonic antigen; P‐AMY, pancreatic amylase; PDAC, pancreatic adenocarcinoma.

Significance levels: *p < 0.05; **p < 0.001; else not significant.

^†^ Continuous variables are expressed as median (IQR) and categorical variables as *n* (%).

^‡^ BMI is the weight in kilograms divided by the square of the height in meters.

^§^ Alcohol consumption was defined as follows: light drinker, <20 g ethanol /day; moderate, 20–60 g/day; and heavy, ≥60 g/day.

### Quantitative analysis

**FIGURE 4:**
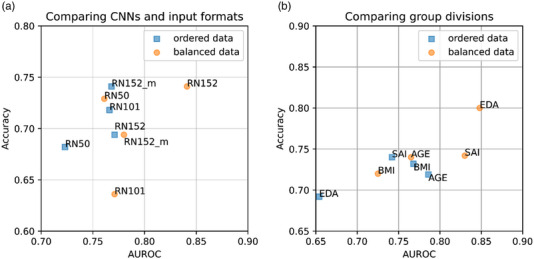
Comparing the mean areas under the receiver operating characteristic curve (AUROCs) and accuracies. (a) Comparing convolutional neural networks (CNNs) and input formats. The mean AUROCs and accuracies of the five different test groups are plotted for ResNet50, ResNet101, and ResNet152 using the ordered or balanced chronology of the sessions datasets. Different versions of ResNet with the larger affixed numbers are deeper and perform better. ResNet152 had a higher AUROC than ResNet152_m. (b) Comparing datasets of different group division manners. The mean AUROCs and accuracies of the five different test groups are plotted for ResNet152 using all datasets. With the balanced endoscopists' diagnostic accuracy (EDA) dataset ResNet152 performed better than with the ordered EDA. The pair of ordered and balanced self‐reported alcohol intake (SAI) datasets showed a similar tendency. The results using the body mass index (BMI) and age (AGE) datasets were the opposite. RN50, ResNet50; RN101, ResNet101; RN152, ResNet152; RN152_m, ResNet152 using the monotone images.

In Figure 4a, the mean AUROCs and accuracies over the five different test groups, resulting from 5‐fold cross‐validation, of both CHR ordered and balanced datasets are plotted for ResNet50, ResNet101, and ResNet152. With the CHR datasets, the larger the affixed number was, the better the AUROC was, as expected. Additionally, ResNet152_m stands for the case where monochrome images, instead of quasi‐RGB images, were used for ResNet152. Notably, replacing monotone images with quasi‐RGB images improved the AUROC of ResNet152.

The average scores of ResNet152 over the five different test groups of individual datasets are found in Table 2, while Figure 4b shows the plots of the AUROCs and accuracies. As a whole, the AUROC, accuracy, sensitivity, and PPV all had high values, suggesting that patients with PDAC were correctly diagnosed with high probability by the retrained ResNet152. However, the NPV and specificity tended to be low. In the balanced datasets, sensitivity, PPV, and NPV were high, but specificity was low, which means somewhat ResNet152 was prone to over‐diagnosing AIP as PDAC. ResNet152 performed much better with the balanced EDA than with the ordered EDA, resulting in a low *P*‐value. This may mean that image features that are recognized by endoscopists actually correlate with important image features extracted by ResNet152, i.e., the training (and validation) and test data share image features meaningful to ResNet152 in the balanced EDA. Meanwhile, the pair of ordered and balanced SAI datasets showed a similar tendency with a high *P*‐value. In contrast, the results with the BMI and AGE datasets showed the opposite, so BMI and age did not seem to correlate with accessible key image features by ResNet152. All scores of ResNet152 over the five different test groups for each dataset are found in Table s1.

**TABLE 2:** Comparison of performance between ResNet152 and endoscopists.

		AUROC		Accuracy		Sensitivity		Specificity		PPV		NPV	
			*p*		*p*		*p*		*p*		*p*		*p*
**CHR**	Ordered	0.77	0.367	0.69	0.359	0.75	0.719	0.53	0.603	0.81	0.355	0.48	0.436
	Balanced	0.84		0.74		0.79		0.62		0.86		0.59	
**AGE**	Ordered	0.79	0.804	0.72	0.796	0.85	0.530	0.40	0.891	0.79	0.937	0.43	0.774
	Balanced	0.77		0.74		**0.89**		0.37		0.79		0.50	
**BMI**	Ordered	0.77	0.624	0.73	0.863	0.84	0.971	0.45	0.873	0.80	0.848	0.71	0.299
	Balanced	0.73		0.72		0.84		0.42		0.80		0.46	
**SAI**	Ordered	0.74	0.433	0.74	0.953	0.84	0.810	0.48	0.800	0.83	0.978	0.55	0.915
	Balanced	0.83		0.74		0.82		0.54		0.83		0.53	
**EDA**	Ordered	0.65	0.086	0.69	0.195	0.80	0.571	0.41	0.237	0.78	0.123	0.59	0.491
	Balanced	**0.85**		**0.80**		**0.87**		**0.63**		**0.87**		**0.73**	
**Endoscopists** ^†^	All			0.63		0.68		0.50		0.78		0.37	
	Experts			0.65		0.68		0.56		0.80		0.42	
	Novices			0.57		0.67		0.42		0.75		0.32	

Abbreviations: AGE, age; AUROC, the area under the receiver operating characteristic curve; BMI, body mass index; CHR, chronology of the sessions; EDA, endoscopists' diagnostic accuracy; NPV, negative predictive value; PPV, positive predictive value; SAI, self‐reported alcohol intake.

Continuous variables are expressed as average.

^†^ Endoscopists were classified as experts or novices based on their qualifications as specialists granted by the Japan Gastroenterological Endoscopy Society.

On average, the experts outperformed the novices in all measures, while ResNet152 outperformed the experts with all datasets in all measures except for specificity.

### Qualitative analysis

**FIGURE 5:**
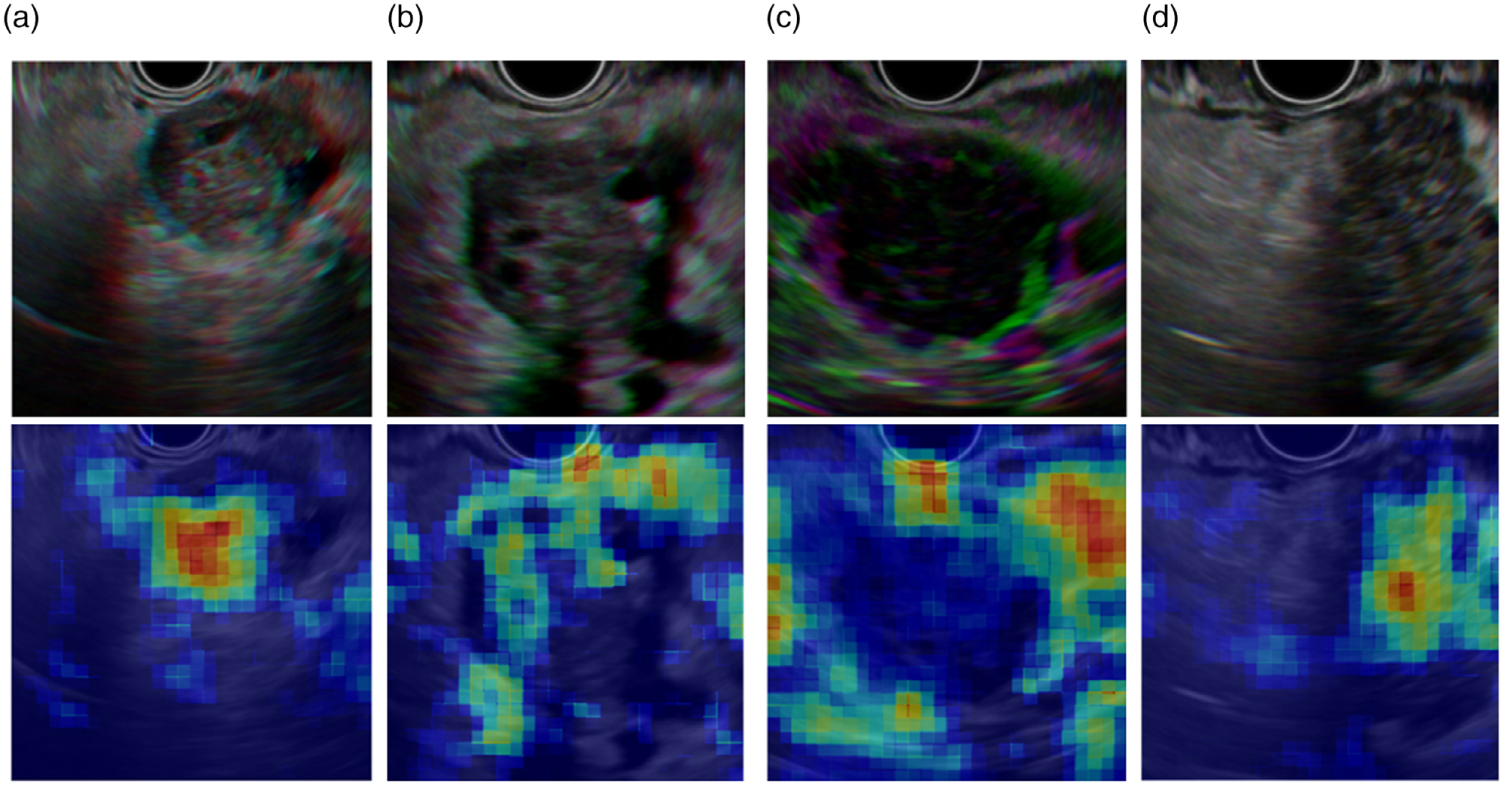
Visualizing neural network predictions. The retrained ResNet152 is visualized via the technique of occlusion. The images are examples of quasi‐RGB images and the corresponding occlusion heatmap analysis: examples of pancreatic ductal adenocarcinoma (a, b) and autoimmune pancreatitis (c, d) results, with the quasi‐RGB image at the top and the occlusion heatmap analysis at the bottom. In the occlusion heatmap analysis, red corresponds to the relevant pixels for the correct predictions while blue corresponds to irrelevant pixels.

To visualize the CNNs’ predictions, we adopted the technique of occlusion. Captum,^21^ which is under the ecosystem of PyTorch, was employed. Captum finds relevant pixels perturbating input images locally and then measures how the outputs of CNNs change. The images in Figure 5 were made by relaying heatmaps of Captum directly to Matplotlib,^22^ where red and blue correspond to relevant and irrelevant pixels, respectively; ResNet152 discriminated lesion‐related areas.

## DISCUSSION

In this study, we established a unique cross‐validation framework to evaluate the training quality of CNNs in relation to varied data splits, aiming to improve deep learning methods in differentiating EUS images of PDACs and AIPs, particularly localized or tumor‐forming AIPs. As a main CNN, we selected ResNet152, which is one of the most popular CNNs for image classification. The results showed that after applying the ensemble method, ResNet152 was superior to human experts in almost all metrics and data splits. Interestingly, the AUROC and accuracy were the highest when the balanced EDA dataset was used.

Even when great care is taken to correctly diagnose PDAC and not over‐diagnose AIP, misdiagnoses may still occur due to human error, which may be avoided by using deep learning. In fact, Marya et al. have successfully differentiated between PDAC and AIP by applying CNNs to EUS images.^12^ However, in our field, we most need the diagnostic ability to differentiate PDACs from tumor‐forming pancreatitis, such as localized or tumor‐forming AIPs, which are difficult to diagnose with imaging. Although training CNNs usually requires a large amount of data, we only had access to limited data since localized AIP is rare. To overcome this problem, we applied widely practiced methods, including transfer learning, data augmentation, K‐fold cross‐validation, and the ensemble method. Additionally, we introduced a unique method of generating quasi‐RGB images from different frames of EUS videos, aiming at augmenting the dataset and processing the video frames as temporal data.

Any machine learning prediction works well when the training (and validation) and test data share accessible common features. As far as ResNet152 is concerned, the endoscopists’ diagnostic performance gave a notable index of differentials from the image features, and the balanced dataset according to that index resulted in the best performance. This does not contradict the fact that CNNs show similar perceptive abilities as human vision that enable certain CNNs to transfer image styles or even generate images. The results suggest that the dataset should be prepared without any human‐vision bias, similar to previous research that concluded that creating heterogeneous training data is important.^23^

Marya et al. reported an EUS‐CNN model for diagnosing AIPs, including both diffuse and localized types,^12^ which achieved excellent diagnostic ability. Although our model showed better diagnostic ability than endoscopists, the low specificity means that our model tended to over‐diagnose AIPs as PDACs. Tonozuka et al. created an EUS‐computer‐assisted diagnosis system that showed good diagnostic ability in detecting pancreatic tumors.^15^ However, they indicated that their model may more likely misdiagnose tumor‐forming chronic pancreatitis (CP) as PDAC compared with non‐tumor‐forming CP. Basu et al. reported a unique model for detecting gallbladder cancer (GBC) using ultrasound images that consists of two stages: detecting the gallbladder and classifying GBCs.^24^ Applying such a method and focusing on regions of interest may improve our results.

The purpose of this study is to investigate efficient learning methods when the data sample obtained for rare diseases is small. Hence, the present study is not yet ready for clinical application and has some limitations. First, our dataset consisted of EUS images of rare diseases at a single institution. Further analysis using datasets with a larger number of patients from multiple facilities would reduce training data bias and enable CNNs to achieve further generalization. Second, there were only two conditions in the present study (AIP and PDAC). We hope to conduct another research on the clinical application of CNNs to EUS image analysis that covers more pancreatic diseases and normal conditions.

In conclusion, we established a unique cross‐validation framework in search of effective data‐splitting methods in training CNNs and confirmed through numerous experiments that CNNs outperformed humans in differentiating AIPs from PDACs. We hope that preparing datasets with fewer human‐vision biases and using quasi‐RGB images will improve the training environments of CNNs to diagnose various diseases on EUS videos.

## CONFLICT OF INTEREST STATEMENT

None.

## Supporting information


**Table S1** All scores of ResNet152 over the five different test groups for each dataset.
AGE, age; AUROC, the area under the receiver operating characteristic curve; BMI, body mass index; CHR, chronology of the sessions; EDA, endoscopists' diagnostic accuracy; NPV, negative predictive value; PPV, positive predictive value; SAI, self‐reported alcohol intake.
