# Supplementary material for: Differentiating localized autoimmune pancreatitis and pancreatic ductal adenocarcinoma using endoscopic ultrasound images with deep learning
Source: DEN Open. 2024 Mar 2;4(1):e344. doi: 10.1002/deo2.344 (PMC10908399; doi:10.1002/deo2.344)
Supplement: Supplementary file 1 — Table S1 All scores of ResNet152 over the five different test groups for each dataset. AGE, age; AUROC, the area under the receiver operating characteristic curve; BMI, body mass index; CHR, chronology of the sessions; EDA, endoscopists' diagnostic accuracy; NPV, negative predictive value; PPV, positive predictive value; SAI, self‐reported alcohol intake. [file DEO2-4-e344-s001.docx]

**Supplementary Tables**

Supplementary Table 1. All scores of ResNet152 over the five different test groups for each dataset

| CHR_ordered | test1 | test2 | test3 | test4 | test5 | average |
| --- | --- | --- | --- | --- | --- | --- |
| AUROC | 0.800 | 0.833 | 0.833 | 0.867 | 0.519 | 0.771 |
| accuracy | 0.647 | 0.647 | 0.765 | 0.765 | 0.647 | 0.694 |
| sensitivity | 0.583 | 0.667 | 0.917 | 0.833 | 0.769 | 0.754 |
| specificity | 0.800 | 0.600 | 0.400 | 0.600 | 0.250 | 0.530 |
| ppv | 0.875 | 0.800 | 0.786 | 0.833 | 0.769 | 0.813 |
| npv | 0.444 | 0.429 | 0.667 | 0.600 | 0.250 | 0.478 |

| CHR_balanced | test1 | test2 | test3 | test4 | test5 | average |
| --- | --- | --- | --- | --- | --- | --- |
| AUROC | 0.923 | 0.767 | 0.900 | 0.867 | 0.750 | 0.841 |
| accuracy | 0.667 | 0.706 | 0.882 | 0.765 | 0.688 | 0.741 |
| sensitivity | 0.538 | 0.917 | 1.000 | 0.750 | 0.750 | 0.791 |
| specificity | 1.000 | 0.200 | 0.600 | 0.800 | 0.500 | 0.620 |
| ppv | 1.000 | 0.733 | 0.857 | 0.900 | 0.818 | 0.862 |
| npv | 0.455 | 0.500 | 1.000 | 0.571 | 0.400 | 0.585 |

| BMI_ordered | test1 | test2 | test3 | test4 | test5 | average |
| --- | --- | --- | --- | --- | --- | --- |
| AUROC | 0.677 | 0.683 | 0.900 | 0.767 | 0.812 | 0.768 |
| accuracy | 0.611 | 0.647 | 0.824 | 0.765 | 0.812 | 0.732 |
| sensitivity | 0.692 | 0.583 | 0.917 | 1.000 | 1.000 | 0.838 |
| specificity | 0.400 | 0.800 | 0.600 | 0.200 | 0.250 | 0.450 |
| ppv | 0.750 | 0.875 | 0.846 | 0.750 | 0.800 | 0.804 |
| npv | 0.333 | 0.444 | 0.750 | 1.000 | 1.000 | 0.706 |

| BMI_balanced | test1 | test2 | test3 | test4 | test5 | average |
| --- | --- | --- | --- | --- | --- | --- |
| AUROC | 0.538 | 0.783 | 0.767 | 0.600 | 0.938 | 0.725 |
| accuracy | 0.667 | 0.706 | 0.765 | 0.588 | 0.875 | 0.720 |
| sensitivity | 0.923 | 0.750 | 0.750 | 0.750 | 1.000 | 0.835 |
| specificity | 0.000 | 0.600 | 0.800 | 0.200 | 0.500 | 0.420 |
| ppv | 0.706 | 0.818 | 0.900 | 0.692 | 0.857 | 0.795 |
| npv | 0.000 | 0.500 | 0.571 | 0.250 | 1.000 | 0.464 |

| AGE_ordered | test1 | test2 | test3 | test4 | test5 | average |
| --- | --- | --- | --- | --- | --- | --- |
| AUROC | 0.723 | 0.700 | 0.833 | 0.717 | 0.958 | 0.786 |
| accuracy | 0.722 | 0.588 | 0.588 | 0.824 | 0.875 | 0.719 |
| sensitivity | 0.923 | 0.750 | 0.833 | 0.917 | 0.833 | 0.851 |
| specificity | 0.200 | 0.200 | 0.000 | 0.600 | 1.000 | 0.400 |
| ppv | 0.750 | 0.692 | 0.667 | 0.846 | 1.000 | 0.791 |
| npv | 0.500 | 0.250 | 0.000 | 0.750 | 0.667 | 0.433 |

| AGE_balanced | test1 | test2 | test3 | test4 | test5 | average |
| --- | --- | --- | --- | --- | --- | --- |
| AUROC | 0.554 | 0.917 | 0.883 | 0.783 | 0.688 | 0.765 |
| accuracy | 0.722 | 0.882 | 0.824 | 0.647 | 0.625 | 0.740 |
| sensitivity | 0.846 | 1.000 | 0.917 | 0.917 | 0.750 | 0.886 |
| specificity | 0.400 | 0.600 | 0.600 | 0.000 | 0.250 | 0.370 |
| ppv | 0.786 | 0.857 | 0.846 | 0.688 | 0.750 | 0.785 |
| npv | 0.500 | 1.000 | 0.750 | 0.000 | 0.250 | 0.500 |

| SAI_ ordered | test1 | test2 | test3 | test4 | test5 | average |
| --- | --- | --- | --- | --- | --- | --- |
| AUROC | 0.954 | 0.583 | 0.833 | 0.883 | 0.458 | 0.742 |
| accuracy | 0.833 | 0.647 | 0.765 | 0.706 | 0.750 | 0.740 |
| sensitivity | 0.846 | 0.917 | 0.750 | 0.667 | 1.000 | 0.836 |
| specificity | 0.800 | 0.000 | 0.800 | 0.800 | 0.000 | 0.480 |
| ppv | 0.917 | 0.688 | 0.900 | 0.889 | 0.750 | 0.829 |
| npv | 0.667 | 0.000 | 0.571 | 0.500 | 1.000 | 0.548 |

| SAI_balanced | test1 | test2 | test3 | test4 | test5 | average |
| --- | --- | --- | --- | --- | --- | --- |
| AUROC | 0.862 | 0.833 | 0.833 | 0.933 | 0.688 | 0.830 |
| accuracy | 0.667 | 0.765 | 0.765 | 0.765 | 0.750 | 0.742 |
| sensitivity | 0.846 | 0.750 | 0.750 | 0.917 | 0.833 | 0.819 |
| specificity | 0.200 | 0.800 | 0.800 | 0.400 | 0.500 | 0.540 |
| ppv | 0.733 | 0.900 | 0.900 | 0.786 | 0.833 | 0.830 |
| npv | 0.333 | 0.571 | 0.571 | 0.667 | 0.500 | 0.529 |

| EDA_ ordered | test1 | test2 | test3 | test4 | test5 | average |
| --- | --- | --- | --- | --- | --- | --- |
| AUROC | 0.631 | 0.767 | 0.917 | 0.517 | 0.438 | 0.654 |
| accuracy | 0.778 | 0.765 | 0.824 | 0.529 | 0.562 | 0.692 |
| sensitivity | 0.923 | 1.000 | 0.917 | 0.500 | 0.667 | 0.801 |
| specificity | 0.400 | 0.200 | 0.600 | 0.600 | 0.250 | 0.410 |
| ppv | 0.800 | 0.750 | 0.846 | 0.750 | 0.727 | 0.775 |
| npv | 0.667 | 1.000 | 0.750 | 0.333 | 0.200 | 0.590 |

| EDA_balanced | test1 | test2 | test3 | test4 | test5 | average |
| --- | --- | --- | --- | --- | --- | --- |
| AUROC | 0.815 | 0.767 | 0.983 | 0.883 | 0.792 | 0.848 |
| accuracy | 0.778 | 0.647 | 0.941 | 0.824 | 0.812 | 0.800 |
| sensitivity | 1.000 | 0.750 | 1.000 | 0.750 | 0.833 | 0.867 |
| specificity | 0.200 | 0.400 | 0.800 | 1.000 | 0.750 | 0.630 |
| ppv | 0.765 | 0.750 | 0.923 | 1.000 | 0.909 | 0.869 |
| npv | 1.000 | 0.400 | 1.000 | 0.625 | 0.600 | 0.725 |

AUROC, the area under the receiver operating characteristic curve; PPV, positive predictive value; NPV, negative predictive value; CHR, chronology of the sessions; BMI, body mass index; AGE, age; SAI, self-reported alcohol intake; EDA, endoscopists diagnostic accuracy.
